# The value of immunotherapy in children with initial short-term frequent seizures

**DOI:** 10.3389/fneur.2022.948727

**Published:** 2022-09-01

**Authors:** Yongheng Zhao, Jun Li, Liang Gao, Xiaofan Yang, Haiqing Zhao, Yumei Li, Li Su, Xiaoyu Zhao, Hao Ding, Baomin Li

**Affiliations:** ^1^Department of Pediatrics, Qilu Hospital of Shandong University, Jinan, China; ^2^Cheeloo College of Medicine, Shandong University, Jinan, China

**Keywords:** autoimmune epilepsy, autoimmune antibody negativity, immunotherapy for epilepsy, antibody prevalence in epilepsy (APE) score, autoimmune encephalitis

## Abstract

This study aimed to discuss clinical characteristics, therapy, and antibody prevalence in epilepsy (APE) score for short-term, frequent epileptic seizures in children who are autoimmune-antibody negative and respond well to immunotherapy. The clinical characteristics, imaging manifestations, electrophysiology, and effective treatment plan of 9 children who met the above criteria were retrospectively analyzed in the Pediatric Neurology Department of Qilu Hospital at Shandong University from June 2019 to December 2021. All 9 patients (6 boys, 3 girls; aged 13 months−11 years and 5 months, median 3.5 years) had acute-onset seizures within 3 months. All had previous normal growth/development with no family history of disease. Seizure types were focal motor seizures (6), generalized tonic-clonic seizures (2), and generalized secondary-to-focal (1); occurred >10 times/day; and lasted <1 min/episode. Formal treatment with ≥2 types of antiseizure medicine (ASM) achieved an unsatisfactory effect. Cranial magnetic resonance imaging showed an abnormal result in 1 case. The APE score was ≥4 in 3 cases and <4 in 6 cases. All patients experienced symptomatic relief with immunotherapy; subsequently, 8 patients were free of recurrence and 1 had significantly reduced seizure frequency. Autoimmune antibody screening is recommended for children who were previously well and have acute-onset epilepsy; high frequency, short-duration seizures; no good response to 2 types of ASM; and other etiologic factors excluded, even with APE score <4. Even with negative autoimmune antibody results, the possibility of autoimmune epilepsy should be considered for urgent initiation of immunotherapy, which can achieve good results.

## Introduction

The correlation between epilepsy and immunity is well established. Similar to West syndrome, Landau–Kleffner syndrome, and other diseases, immunotherapy is the first-line treatment for autoimmune epilepsy. Autoimmune epilepsy (AE), first proposed by Levite in 2002 (1), describes epilepsy in which a series of immune cells and antibodies are associated. Until 2017, the International League Against Epilepsy (ILAE) formally listed the immune factor as 1 of the 6 etiologic factors of epilepsy, and formally proposed the definition of *autoimmune epilepsy*: Epilepsy is caused by immune dysfunction, and the key symptom of the immune dysfunction is a seizure episode (2). Research reported that the HMGB1/cxcl12-mediated immunity and T helper cell 17 activation may play a key role in the pathogenesis of AE (3).

In recent years, the popularization of autoimmune antibody screening has consistently improved the diagnostic rate of autoimmune encephalitis (4). As the antibody of autoimmune encephalitis is the same as that of AE, and both can cause epileptic seizures and cognitive mental disorder, the tendency is to misuse the term “*autoimmune epilepsy*.” Recent studies on patients with encephalitis recovering from treatment reported that the epileptic seizures in autoimmune encephalitis were gradually reduced, the incidence of long term epileptic seizure was low, and the difference between *seizure* and *epilepsy* was significant (5, 6).

In 2020, the ILAE clearly differentiated the conceptual difference between “*acute symptomatic seizures secondary to autoimmune encephalitis*” and “*autoimmune-associated epilepsy*” (7). The former is in the process of autoimmune encephalitis in the occurrence of epilepsy, through immune targeted treatment, the main symptoms of autoimmune encephalitis is relieved at the same time, the seizures can be completely controlled, and in most cases, can be completely out of use antiseizure medicines (ASMs) finally. AE is also different from autoimmune-associated epilepsy. Autoimmune-associated epilepsy refers to chronic epilepsy secondary to autoimmune brain disease, where some immune-mediated attack can become a chronic brain disease in patients. Although ASMs and immunotherapy are invalid, there is evidence that cytotoxic T cells play an important role in pathogenic mechanism; it leads to the death of neurons, and the nervous system autoantibodies are more of an immune response by-product that do not have a direct pathogenic effect. Therefore, the etiology of autoimmune-associated epilepsy emphasizes the pathogenic role of brain structural factors, whereas, AE only emphasizes that immune factors play a leading role in its etiology and does not include structural factors. Although the therapeutic effect of ASMs is not good for AE, the effect of immunotherapy is better. At present, there is still no clear diagnostic criteria for AE and the negative results of serum and cerebrospinal fluid antibodies do not exclude its possibility. This study retrospectively reviewed 9 children with epileptic seizures who had short-term recurrent seizures with autoimmune antibody negativity and responded well to immunotherapy. The patients were analyzed for clinical manifestations, auxiliary examination results, the effect of immunotherapy and APE score (8) (Table 1).

**Table 1:** Antibody prevalence in epilepsy (APE) score.

**Clinical manifestations**	**Scoring**
New-onset, rapid progressive mental symptoms more than 1–6 weeks or new-onset epileptic seizure (evaluated within 1 year)	1
Neuropsychiatric changes; Restlessness, aggression, emotional instability	1
Autonomic dysfunction [persistent atrial tachycardia or bradycardia, erect hypotension (systolic blood pressure drop ≥20 mm Hg or diastolic blood pressure drop ≥10 mm Hg within 3 min of standing), hyperhidrosis, persistent unstable blood pressure, ventricular tachycardia, or cardiac arrest]	1
Viral prodrome (runny nose, sore throat, low fever), no potential systemic malignancy	2
Facial dystonia or faciobrachial dystonia	2
No response to at least 2 types of AEDs	2
CSF results showing inflammatory changes (CSF protein > 50 mg/dL and/or lymphocytes > 5/dL when CSF RBC count <1,000/mm^3^)	2
Brain MRI showing signal changes consistent with limbic encephalitis (Altered T2/FLAIR signal in the Medial temporal lobe)	2
Presence of potential malignancies (excluding cutaneous squamous cell carcinoma and basal cell carcinoma)	2

CSF, cerebrospinal fluid; MRI, magnetic resonance imaging; RBC, red blood cell.

## Materials and methods

### Study population

The study included 9 children considered to have AE. All patients were hospitalized in the Department of Pediatrics, Qilu Hospital, Shandong University, from June 2019 to December 2021. The inclusion criteria were as follows: (1) course of disease <3 months; (2) epileptic seizures occurring with high frequency, with an average daily seizure frequency of >10 times and each seizure duration being relatively short—usually <1 min; (3) negative serum + cerebrospinal fluid (CSF) neural autoimmune antibody examination, including the antibodies NMDA-R-Ab, LGI1-Ab, CASPR2-Ab, GABAB-R-Ab, GAD65-Ab, AMPA1-R-Ab, AMPA2-R-Ab, and anti-YO antibody; (4) development of mental motor was normal before onset, no family disease history, no history of epilepsy caused by tumor, cerebrovascular disease, encephalitis, trauma, or metabolic disease and others; (5) absence of definite radiographic abnormalities; and (6) no good response to ≥2 types of ASM and later acceptance of systemic immunotherapy. Patients were 6 boys and 3 girls, age 13 months−11 years and 5 months (median 3.5 years).

### Methods

Patients were assessed for their clinical background, including medical history, symptoms, physical signs, and CSF analysis; imaging results; electrophysiology; serum antibody concentrations; and auxiliary examinations, including CSF autoimmune antibody testing; APE score; effective treatment plan; follow-up status; and prognosis. Each child was followed up for 1, 3, and 6 months after immunotherapy. All patients were followed up for at least 6 months.

## Results

### Epileptic seizure conditions

Among the nine patients, seizure types were focal motor seizures (6 patients, 66.7%), generalized tonic-clonic seizures (two patients, 22.2%), and generalized secondary-to-focal (1 patient, 11.1%), and 1 patient (11.1%) had special manifestations, such as paroxysmal limb pain and panic. The epileptic seizure emerged with high frequency, with the average daily seizure frequency >10 times, and the duration of each seizure was relatively short, usually <1 min. The use of ≥2 types of ASM did not provide a satisfactory treatment effect.

### Auxiliary examination results

All nine patients underwent CSF examination and cytology in addition to routine, biochemical, immune, and culture examinations, with no abnormalities reported. Examination of serum + CSF neural autoimmune encephalitis antibody was negative, which included NMDA-R-Ab, LGI1-Ab, CASPR2-Ab, GABAB-R-Ab, GAD65-Ab, AMPA1-R-Ab, and AMPA2-R-Ab. In addition, demyelinating antibody (anti-MOG antibody IgG, anti-AQP4 antibody IgG, anti-MBP antibody IgG, anti-amphiphysin antibody IgG, anti-Hu antibody IgG, anti-Ri antibody IgG, anti-SOX1 antibody IgG) was negative.

**Figure 1:**
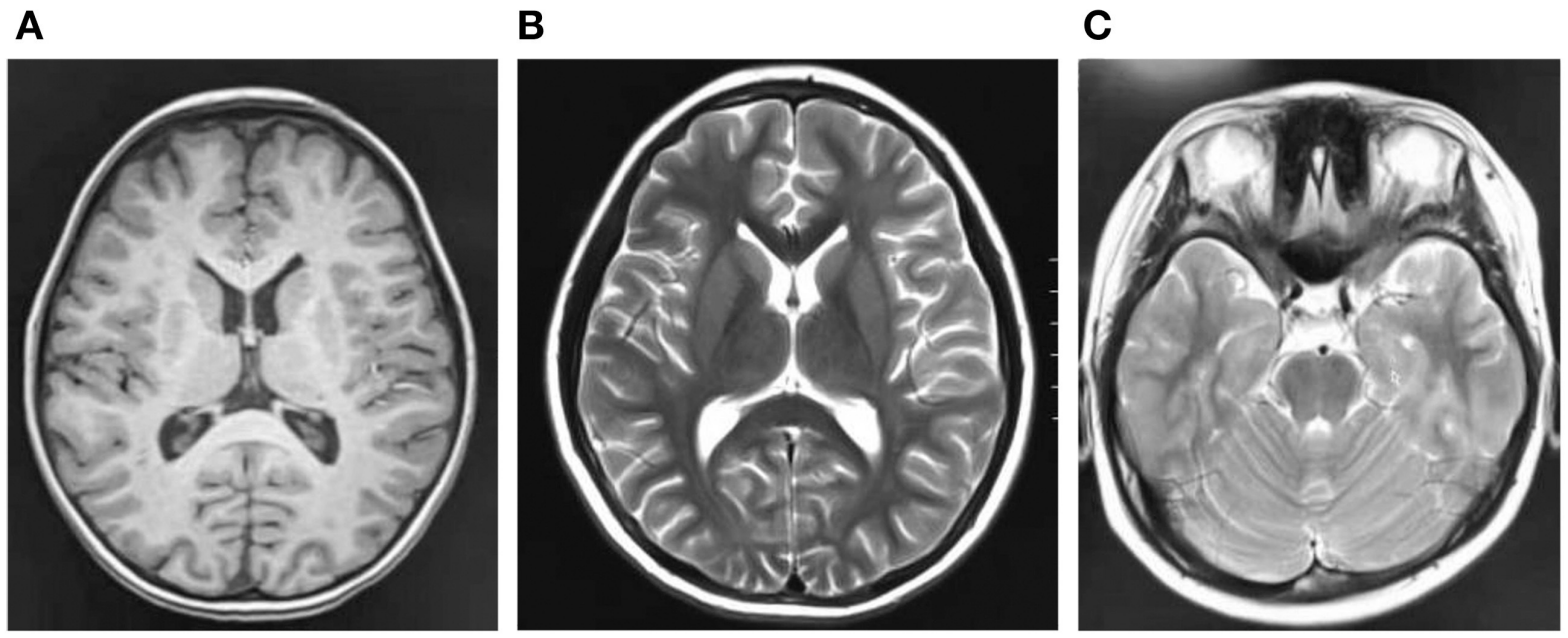
**(A–C)** Are craniocerebral magnetic resonance imaging of some children, and the results are negative.

**Figure 2:**
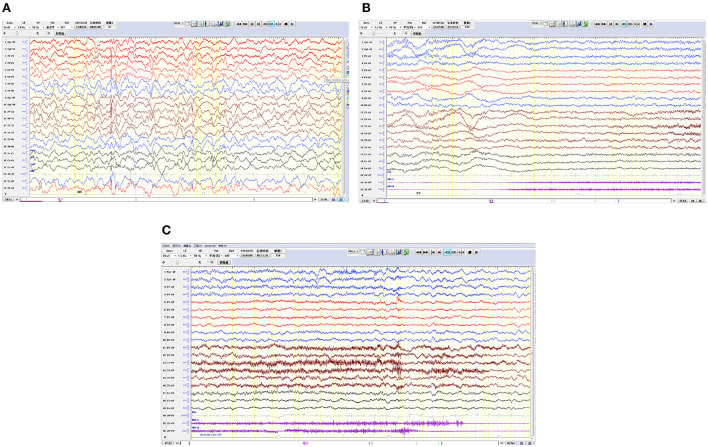
Electroencephalograms for 3 pediatric patients. **(A)** The right hemisphere is dominated by multifocal spike waves and slow spike waves. **(B,C)** Generalized decreased voltage. In the bilateral frontal pole and frontal and anterior temporal regions, a fast wave is actively released with a low amplitude, lasting for 20 s, and in the late stage of onset, 1–3 Hz δ is actively released in the temporal region and accompanied by a large amount of electromyography pseudo error.

Cranial magnetic resonance imaging (MRI) showed an abnormal result in 1 case; results were normal for the other eight cases (Figure 1). All nine patients demonstrated an electroencephalograph (EEG) abnormality (Figure 2). The specific examination results are shown in Table 2.

**Table 2:** Clinical background data for all study patients.

**Number of cases**	**Sex**	**Age**	**Manifestation of epileptic seizure**	**Craniocerebral MRI**	**EEG**	**Antibody result (serum/CSF)**	**CSF result**	**APE score**	**ASMs**	**Immunotherapy**	**Effect of immunotherapy**
1	Female	3 years 2 months	Focal seizure	No abnormality	Right central, temporalis media-onset focal seizure	-/-	-	5	OXC, VPA, CZP	IVIG	Good
2	Male	4 years 4 months	Focal seizure	No abnormality	Dominant in the right hemisphere and have extensive spike wave, spike slow wave, multi spikes slow wave, and fast wave rhythm	-/-	-	3	OXC, VPA	IVIG	Completely no attack
3	Male	1 year 1 month	Generalized seizure	No abnormality	During the sleeping period, the frontal, central, and midline areas of the bilateral atypical sharp wave, slow wave compound small sharp wave	-/-	-	5	OXC, VPA	IVIG	Completely no attack
4	Male	2 years 5 months	Generalized seizure	No abnormality	In sleeping period, bilateral frontal, central, parietal, Fz, Cz spike wave, and slow spike wave	-/-	-	3	OXC, VPA	IVIG+ IVMP	Completely no attack
5	Male	3 years 6 months	Focal seizure	The temporal horn of the right ventricle is slightly enlarged	Focal onset in the right middle temporal region	-/-	-	2	OXC, VPA, CZP	IVIG+ IVMP	Completely no attack
6	Male	11 years 5 months	Focal seizure	No abnormality	During sleeping period, regular rhythm of spike wave, slow spike wave, multiple-spike wave, low amplitude fast wave was found in frontal lobe	-/-	-	3	VPA, LEV, CZP, LCM	IVIG	Completely no attack
7	Male	11 years	Focal seizure	No abnormality	The left central region began with focal episodes of seizures with consciousness	-/-	-	3	OXC, LCM, CBZ	IVIG	Completely no attack
8	Female	1 year 7 months	Focal seizure	No abnormality	The anterior head is mainly spike wave, slow spike wave, multi spike slow wave, sharp slow wave, sharp slow wave, irregular slow wave, and fast wave. Obvious in left side	-/-	-	5	OXC, LCM	IVIG	Completely no attack
9	Female	4 years	Focal seizure secondary to generalized seizure secondary	No abnormality	In the right central, occipital, and middle temporal regions, spike, and slow spike were released. During sleeping period, the right hemisphere was dominant, and the extensive slow spike waves, multi spike slow wave, slow spike slow wave, and 15–21 Hz fast waves were regularly released	-/-	-	3	VPA, LEV, CZP, OXC	IVIG	Effective

The number of seizure episodes after immunotherapy decreased ≥50 and <75% was considered effective; decrease ≥ 50 and <100% was considered good; decrease 100% was considered completely no seizure episodes. When the steady-state concentration is reached after 7–10 days of drug treatment, the number of seizure episodes decreased by <50% was considered invalid. AEDS, Anti-epileptic Drugs; APE, Antibody Prevalence in Epilepsy; CZP, Clonazepam; EEG, Electroencephalogram; IVIG, intravenous immunoglobulin; IVMP, Intravenous methylprednisolone; LCM, Lacosamide; CBZ, Carbamazepine; LEV, Levetiracetam; MRI, magnetic resonance imaging; OXC, oxcarbazepine; VPA, Valproic acid. Dose: IVIG 2 g/kg divided over 2–5 days, IVMP 20 mg/kg per day for 3 days followed by Sequential treatment with oral methylprednisolone.

### Immunotherapy results

All nine patients had already tried ≥2 types of ASMs, with poor therapeutic effects—their seizures were not reduced. One month after the first immunotherapy, epileptic seizure did not occur in 4 children, and occasional epileptic seizure occurred in 1. The other four children had obvious decrease in epileptic seizure frequency, and while three of them were administered a second course of immunotherapy, one declined further treatment for personal reasons.

At 3 months after the first immunotherapy, eight patients no longer experienced epileptic seizures. Among these eight patients, three had undergone a second course of immunotherapy 1 month after the first immunotherapy. The one patient who did not accept the second course of immunotherapy experienced reduced epileptic seizure frequency, shorter duration, and reduced intensity of the seizure episodes, and an objectively improved mental status and cognitive function.

At 6 months after the first immunotherapy, eight patients continued to experience freedom from seizures. The other one patient occasionally experienced epileptic seizure, with greatly reduced frequency and duration, and significantly improved cognition and quality of life.

Among the nine patients in our current study, seven were only treated with IVIG, and the remaining two were treated with IVIG combined with IVMP, which all had significant effects. Detailed use of immunotherapy is displayed in Table 2.

### APE score

The APE score was ≥4 in 3 cases and <4 in six cases; however, the score was not correlated with the treatment effect.

## Discussion

Epilepsy is a common chronic brain disease caused by multiple etiologic factors that result in the abnormal discharge of brain neurons and lead to the occurrence of clinical symptoms. Recently, increasingly more studies are focused on the etiology of epilepsy. Until 2017, ILAE officially listed immune factors as 1 of the 6 etiologies of epilepsy (2): structural, genetic, infectious, metabolic, immune, and unknown. The increased diagnosis of autoimmune encephalitis—which has the same anti-neural antibody as autoimmune-associated epilepsy and, in most cases, includes epileptic seizures, resistance to ASM, and other similar characteristics—has led to the widespread misuse of the diagnosis of AE and to the excessive use of ASMs (7, 9).

**Figure 3:**
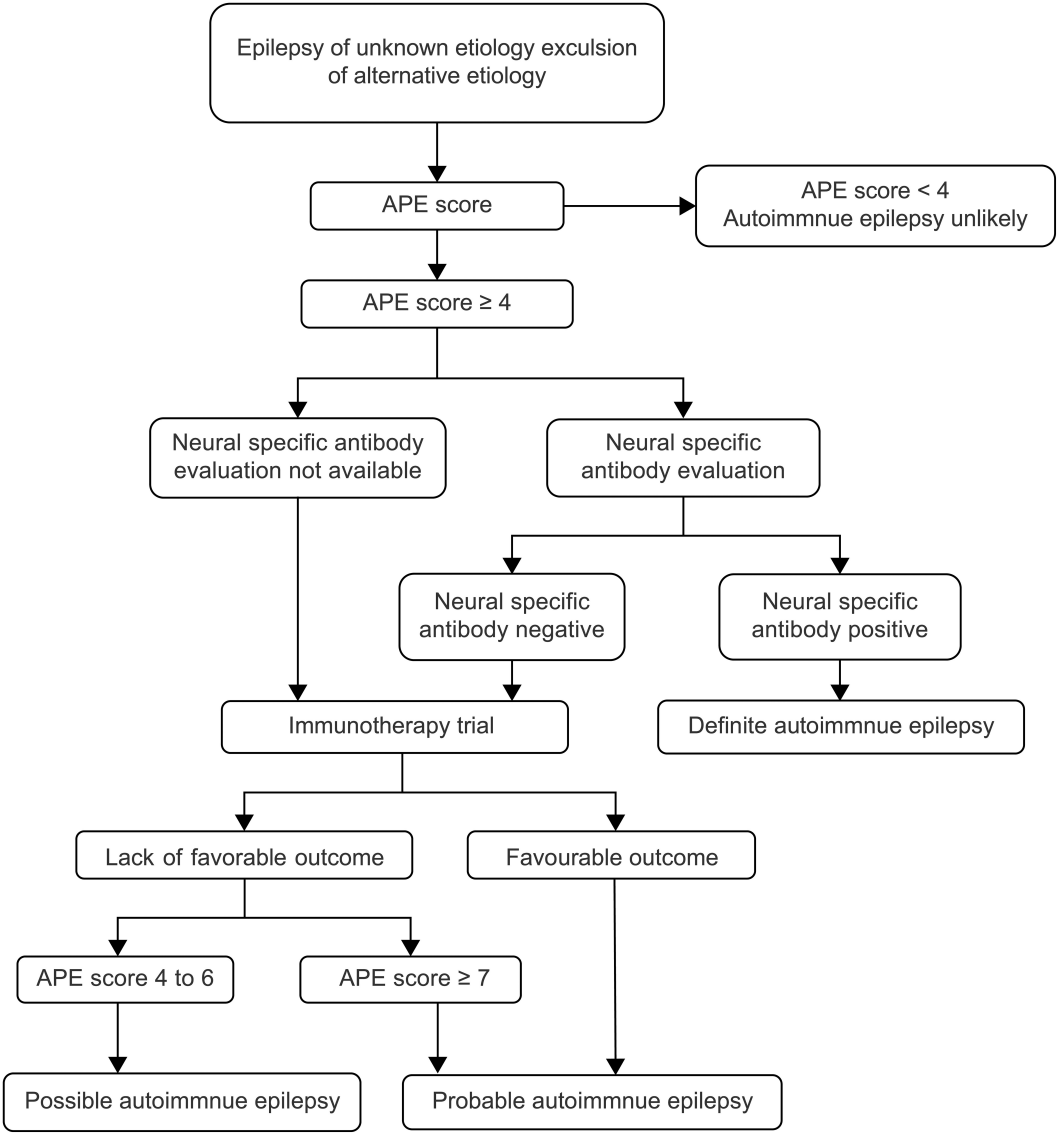
Flow diagram of diagnosis and therapy (15). APE, antibody prevalence in epilepsy.

In July 2020, ILAE made a clear distinction between the two concepts in Epilepsia magazine, describing “acute symptomatic seizures secondary to autoimmune encephalitis” and “autoimmune-associated epilepsy” (7). In fact, the epileptic seizure in autoimmune encephalitis did not respond well to the treatment with multiple ASMs. Most pediatric patients can control the epileptic seizure after achieving control of autoimmune encephalitis, and do not need to take ASMs for a long term, and the possibility of developing epilepsy is relatively small (5, 10, 11). AE is increasingly recognized as a potentially reversible cause of frequent medically refractory seizures and cognitive decline. At present, there is no clear diagnostic standard for AE. Previously, it was generally believed that any disease accompanied by seizures and autoantibodies was AE (12). Therefore, most autoimmune encephalitis is usually classified as AE, regardless of the disease causing the seizure, antibody type, or the definition of epilepsy used (13). This widely used classification is inaccurate and has led to the development of an APE scoring system based on the same clinical perspectives and diagnostic criteria as antibody-associated encephalitis, leading to important selection biases (8). Some scholars believe that the diagnostic criteria of AE can be determined according to APE score, neurospecific antibody, and immunotherapy test (14, 15) (Figure 3). The APE score can be very high in children with autoimmune encephalitis and even epilepsy caused by common encephalitis and abnormal CSF and cranial MRI. At present, there is a great controversy over the use of APE score, and some scholars have proposed that fluorodeoxyglucose (FDG)-positron emission tomography (PET) examination is of great value to supplement the APE score, especially in pediatric patients with negative MRI (16).

Other studies have shown that cytokines or chemokines can be used as alternative biomarkers for diagnosing suspected AE (sAE) (3). Prominent changes in several immune and inflammatory pathways, such as IL-1R1/TLR4, COX-2, TNF-a, and chemokines have been reported within the epileptogenic lesions in clinical studies. Specific drugs or interventions targeting them may decrease established seizures or play a role in antiepileptogenesis effects (17). Currently, inflammation is believed to contribute to the occurrence of epilepsy, and epilepsy seizures have pro-inflammatory activities. AE is one of the common causes of status epilepticus (SE), which damages the blood–brain barrier. Activation of astrocytes, microglia, reactive astrocytes, and other inflammatory mediators, through various pathways, ultimately lead to the influx of calcium ions in neurons, reduce the threshold of epileptic seizures, thus aggravating the onset of SE. Therefore, activation of the immune system can be both the cause and result of seizures (18). The diagnosis of AE is very important—the earlier the diagnosis, the better the prognosis (19). At present, the diagnosis of AE greatly relies on antibody detection and immunotherapy response. When antibody detection is difficult, imaging is particularly important. About 40% of pediatric brain MRI results showed no abnormalities. The abnormal MRI signals are typically in 1 or both medial temporal lobes, and some AE involve structures other than temporal lobes, including basal ganglia, non-limbic cortex, cerebellum, brainstem, and white matter (20). In addition to brain F-FDG-PET examination, whole-body F-FDG-PET can help identify potential tumors and detect metastasis. Studies have found that brain MRI is not always related to brain F-FDG-PET, so these 2 examinations can complement each other (21, 22). Both MRI and PET are valuable tools in identifying and evaluating the progression of autoimmune-related epilepsy. Because F-FDG-PET is usually more sensitive to the brain lesions of AE, it is recommended to perform F-FDG-PET early when the diagnosis of AE is suspected clinically, especially for cases in which MRI is normal or cannot be judged (23). AE on EEG is characterized by multiple focal seizures with repeated sharp slow wave discharge (24).

At present, there are still no clear diagnostic criteria for AE. After a wide literature review and based on with our experience with the children admitted to our hospital who have AE, we believe that AE should be considered in these cases: first, for children with a positive serum or CSF antibody test, after excluding autoimmune encephalitis and with epilepsy seizure as the only manifestation, AE can be considered as a diagnosis; and second, for children with a negative antibody test, the need to meet the following conditions can also be considered to diagnose AE: (1) normal growth and development in the past, sudden-onset of frequent seizures within the past 3 months, short seizure duration, usually <1 min; (2) standard treatment with ≥2 ASMs is not effective; (3) exclusion of intracranial infection, cerebrovascular disease, tumor and other diseases with clear etiology; (4) brain MRI is negative; and (5) after immunotherapy, the frequency of seizures decrease significantly.

In terms of treatment, the addition of immunotherapy can better control seizures than the use of ASMs alone (25). The first-line treatment of AE in children is hormone, human immunoglobulin, and TPE, among which TPE is rarely used in pediatric patients versus adults, especially in the case of restlessness or autonomic nervous instability (14, 26–28). Depending on the response to treatment and the severity of the clinical presentation, combination therapy, usually high-dose intravenous methylprednisolone (IVMP) shock and intravenous immunoglobulin (IVIG), or second-line therapy (rituximab or cyclophosphamide), may be considered. Among the 9 patients in our current study, 7 were only treated with IVIG, and the remaining 2 were treated with IVIG combined with glucocorticoid, all of which had significant effects. According to the different clinical manifestations among pediatric patients, ASMs are still needed to reduce the frequency of epileptic seizures or the duration of epilepsy while waiting for immunotherapy to take effect (29). At present, the latest study indicates that neuroinflammation is a potential target for the treatment of epilepsy, especially for children with drug-resistant and refractory status epilepsy. The experience of anti-cytokine agents and lymphocyte-targeting drugs for the treatment of epilepsy mainly comes from case reports or series. The use of anti-IL-1, anti-IL-6, and anti-CD20 agents in patients with drug-resistant and refractory epileptic status has shown good therapeutic results. Current experience with TNF inhibitors is limited to Rasmussen encephalitis (30). Moreover, our study had some limitations, 1 of which is that the number of cases in this study was small.

Although there is currently no effective pediatric scale to identify the population in need of antibody testing, screening for AE is necessary to identify patients who may be eligible for immunotherapy and to avoid overuse of ASM and excessive psychological stress for parents. Since not all antibodies have been discovered and reported, and not all discovered antibodies can be detected, negative MRI results of antibodies, cerebrospinal fluid, and brain do not exclude the possibility of AE (31, 32). For newly emerged seizures of short duration and high frequency with poor response to ASMs, and the exclusion of other clear causes in pediatric patients, the possibility of AE should be considered, with the relevant antibody screening carried out as soon as possible. Even if results are negative, experimental immunotherapy should be carried out at the earliest.

## Data availability statement

The raw data supporting the conclusions of this article will be made available by the authors, without undue reservation.

## Ethics statement

The studies involving human participants were reviewed and approved by Medical Ethics Committee of Qilu Hospital of Shangdong University. Written informed consent to participate in this study was provided by the participants' legal guardian/next of kin. Written informed consent was obtained from the individual(s), and minor(s)' legal guardian/next of kin, for the publication of any potentially identifiable images or data included in this article.

## Author contributions

YZ, JL, and BL were responsible for the original concept, overall design of the research, and wrote and revised the manuscript. YZ, JL, XY, and BL analyzed the EEG results and diagnosed the patients. LG, HZ, YL, LS, XZ, and HD collected the clinical data and statistical analysis. All authors read and approved the final manuscript.

## Funding

This study was supported by the Foundation of National Key Research and Development Program of China (NO. 2016YFC1306202).

## Conflict of interest

The authors declare that the research was conducted in the absence of any commercial or financial relationships that could be construed as a potential conflict of interest.

## Publisher's note

All claims expressed in this article are solely those of the authors and do not necessarily represent those of their affiliated organizations, or those of the publisher, the editors and the reviewers. Any product that may be evaluated in this article, or claim that may be made by its manufacturer, is not guaranteed or endorsed by the publisher.
